# High expression of CDKN2A is associated with poor prognosis in colorectal cancer and may guide PD-1-mediated immunotherapy

**DOI:** 10.1186/s12885-023-11603-w

**Published:** 2023-11-11

**Authors:** Yuying Dong, Mingming Zheng, Xiaoxuan Wang, Chenyue Yu, Tiantian Qin, Xuning Shen

**Affiliations:** 1 Zhejiang Chinese Medical University, Hangzhou, People’s Republic of China; 2 Affiliated Hospital of Jiaxing University, Jiaxing, Zhejiang People’s Republic of China

**Keywords:** CDKN2A, Colorectal cancer, Programmed death protein 1, Prognostic biomarker, Immunotherapy

## Abstract

**Background:**

Colorectal cancer (CRC) is one of the most common malignancies worldwide. Immunotherapy targeting the programmed death protein 1(PD-1) and its ligand (PD-L1), is a promising treatment option for many cancers, but has exhibited poor therapeutic efficacy in CRC. This study aimed to identify and validate the prognostic value of immune-related genes and PD-1-associated genes for immunotherapy treatment of CRC.

**Methods:**

An extensive analysis of prognostic immune-related DEGs and PD-1-related genes has highlighted CDKN2A as a vital overlapping gene. To further explore its expression in CRC and its prognostic value, we conducted qRT-PCR, Western blot experiments, and consulted various databases. Subsequently, we conducted gene expression analysis, survival and prognostic analysis, enrichment analysis, immune infiltration assessment, and TIDE analysis to assess the significance of CDKN2A.

**Results:**

In CRC, CDKN2A was highly expressed compared to normal tissue. It was found that CDKN2A expression was related to clinicopathological features such as inflammation and tumor stage. Furthermore, a significant correlation was identified between CDKN2A and immune infiltration, specifically involving CD4 T cells, CD8 T cells, and macrophages. The analysis of the GSEA of CRC samples with high CDKN2A expression identified enrichment of genes involved in MYC target-v2 and metabolism pathways. Furthermore, UBE2I, CDK4, CDK6, TP53, and CCND1 were found to be significantly coexpressed with CDKN2A, suggesting a potential role that these gene play in CRC and immunotherapy.

**Conclusions:**

Our study revealed that high CDKN2A expression in CRC is a potentially valuable prognostic biomarker, which may guide PD-1-mediated immunotherapy.

**Supplementary Information:**

The online version contains supplementary material available at 10.1186/s12885-023-11603-w.

## Introduction

Colorectal cancer (CRC) ranks as the third most prevalent malignancy worldwide and stands as the second primary contributor to cancer-related mortality [1]. Conventional therapeutic modalities for CRC encompass surgical intervention, chemotherapy, and radiotherapy, however, most patients are diagnosed at intermediate or advanced stages, resulting in dismal prognosis and poor survival [2, 3]. Therefore, it is crucial to identify novel biomarkers and molecular targets to develop more effective treatment strategies to improve outcomes for patients with intermediate and advanced CRC.

Immunotherapy uses the immune system to target tumor cells and tissues, and can have advantages over chemotherapy and radiotherapy, especially regarding specific targeting of cancer cells [4]. Immune checkpoint inhibitors(ICIs) offer a promising immunotherapeutic approach, and have obtained U.S. Food and Drug Administration (FDA) approval for the treatment of several advanced malignancies [5, 6]. In CRC, immune checkpoint therapy has obtained regulatory approval for the treatment of tumors exhibiting a substantial mutational burden, characterized by deficiencies in mismatch repair (dMMR) mechanisms or high levels of microsatellite instability (MSI-H). However, colorectal cancer (CRC) tumors that display proficient mismatch repair (pMMR), microsatellite stability (MSS), or demonstrate low levels of microsatellite instability (MSI-L; commonly referred to as pMMR–MSI-L tumors) do not manifest a favorable response to immunotherapy [7, 8]. Hence, there is a pressing need for the identification of more dependable biomarkers and molecular targets to enhance the effectiveness of immune checkpoint therapy and optimize treatment outcomes in patients with colorectal cancer.

The immune checkpoint targets, such as Programmed Cell Death 1 (PD-1) and Cytotoxic T Lymphocyte Antigen 4 (CTLA-4), have been extensively investigated in academic research. PD-1 and its corresponding ligand, Programmed Cell Death Ligand 1 (PD-L1), are widely acknowledged as crucial components of immune checkpoints, frequently observed in heightened levels within tumor microenvironments. This immunosuppressive microenvironment helps cancer cells to escape destruction by the immune system [9–11].

The objective of this study was to ascertain the prognostic genes linked to PD-1 and immunity in colorectal cancer. Furthermore, we investigated the correlation between these genes and various clinicopathological aspects of CRC, such as immune infiltration and the immune microenvironment. We successfully identified and validated the expression and prognostic significance of this pivotal gene, and subsequently delved into its biological functionalities. These data may identify novel biomarkers that can guide or improve PD-1-mediated immunotherapy of CRC.

## Materials and methods

### Data acquisition

Transcriptomes files and clinical data related to colorectal cancer were obtained from the TCGA (TCGA-COAD) and GEO databases (GSE39582, GSE24551, GSE18105, GSE40967). Additionally, we retrieved immune-related gene expression data from the ImmPort and InnateDB databases. Furthermore, genes related to PD-1 expression were selected from the GeneCards and NCBI databases. Pan-cancer data were obtained from the UCSC database.

### Clinical tissue samples

According to the Declaration of Helsinki, this study was approved by the Ethics Committee of the First Hospital of Jiaxing. A total of 66 samples of CRC tissue with adjacent normal tissue were collected at the First Hospital of Jiaxing and stored at -80°C. Pathologists and clinicians provided and verified information on relevant clinical features.

The study incorporated the following inclusion criteria: (1) individuals diagnosed with primary colorectal cancer; (2) individuals with a confirmed diagnosis through pathological examination; and (3) individuals who had not undergone preoperative chemotherapy, radiotherapy, or targeted therapy. Conversely, the exclusion criteria encompassed: (1) patients with incomplete or ambiguous pathological data; and (2) patients who had undergone alternative treatments prior to surgery.

### Identification of prognostic immune-related genes in COAD

In this study, we utilized the R- x64–4.1.2 'limma' package to conduct a differential expression analysis of immune-related genes (irDEGs) in COAD. We applied a false discovery rate (FDR) threshold of less than 0.05 and a log-fold change (FC) threshold greater than 1 to identify significant irDEGs. Additionally, we employed a univariate Cox analysis using the 'survival' package to examine the relationship between the expression levels of irDEGs and the survival of COAD patients. We considered irDEGs with a *p* value less than 0.05 as potential prognostic markers. To minimize bias and account for surgical factors, we excluded patients with missing values and those who died within a 30-day follow-up period from our analysis.

### Enrichment analyses and network visualization

In order to examine the biological characteristics of the prognostic irDEGs, we conducted Gene Ontology (GO) and Kyoto Encyclopedia of Genes and Genomes (KEGG) pathway enrichment analyses using the 'clusterProfiler', 'enrichplot', and 'ggplot2' packages [12–14]. Statistical significance was determined using an FDR threshold of 0.05. Additionally, the protein–protein interaction (PPI) network of the genes within this interaction network module was investigated using the STRING database.

### Identifying PD-1-related DEGs and determining their association with prognostic irDEGs in COAD

We retrieved PD-1-related genes from GeneCards and the NCBI database, and conducted a difference analysis to identify PD-1-related DEGs in COAD using the following criteria:|LogFC|> 1 and FDR < 0.05. The application of Venn diagram analysis was subsequently utilized to ascertain the genes that overlapped between the prognostic irDEGs and PD-1-related DEGs. The visualization of this intersection was accomplished through the utilization of the R-package 'Venn'.

### Pancancer analysis of CDKN2A expression

Using the UCSC database, we obtained pancancer expression data, and then extracted the CDKN2A gene expression data for each sample. Significant differential analysis was performed using the log2 (x + 0.001) transformation and the Wilcoxon rank test.

### mRNA expression and the prognostic significance of CDKN2A in COAD

In order to examine the potential correlations between the expression of CDKN2A and the survival outcomes of patients diagnosed with COAD, we conducted an analysis of gene expression profiles utilizing the GEPIA and UALCAN databases. Furthermore, we evaluated the expression of CDKN2A in COAD through the utilization of the R-package 'timeROC', wherein we plotted the receiver operating characteristic curve (ROC), while also calculating the corresponding area under the curve (AUC).

### Quantitative real-time PCR (qRT-PCR) in CRC

We extracted total RNA from CRC samples, reverse transcribed it to cDNA template, and performed RT-qPCR. Gene expression and relative gene expression were analyzed using the 2^−∆Ct^ and 2^−∆∆Ct^ approaches, respectively [15]. The primer sequences used, with β-actin as the endogenous control, are as follows: CDKN2A-F 5ʹ-CAAGATCACGCAAAAACCTCTG-3’; CDKN2A-R 5ʹ-CGACCCTATACACGTTGAACTG-3’; β-actin-F 5ʹ-TGGCACCCAGCACAATGAA-3ʹ; β-actin-R 5ʹ-CTAAGTCATAGTCCGCCTAGAAGCA-3ʹ.

### Western blot

We added a Protease Inhibitor to the RIPA buffer in order to extract proteins from colorectal cancer and its paired normal tissues. Following this, we used the BCA Protein Assay kit to quantify the proteins, and then conducted a 12.5% SDS-PAGE. The proteins were transferred to a 0.2 µm PVDF transfer membrane, which was subsequently incubated with 5% skimmed milk powder for 1.5 h at room temperature. The membrane was then incubated with primary antibodies (β-actin 1:5000, CDKN2A 1:4000, Proteintech) at 4 °C overnight. The PVDF was then incubated with a secondary antibody (HRP-conjugated goat anti-rabbit 1:5000) for 1.5 h, and finally, we used chemiluminescence to detect the protein expression.

### Gene Set Enrichment Analysis (GSEA) of CDKN2A

The significant biological functions and pathways linked to low and high CDKN2A expression were identified using GSEA-4.2.3. Phenotyping was conducted based on the median CDKN2A expression level. The data was analyzed and visualized using the Hallmark and KEGG tools. Significance was determined by an adjusted *p* value of less than 0.05, a normalized enrichment score (|NES|) greater than 1, and a false discovery rate (FDR) below 0.25.

### Correlation between CDKN2A expression and the tumor microenvironment in CRC

The tumor microenvironment (TME) refers to the tissue environment surrounding cancer cells, and includes not only cancer cells but also immune and stromal components. In this study, data were gathered from patients diagnosed with CRC, and the R-'estimate' package was employed to calculate stromal, immune, and ESTIMATE scores. Additionally, an investigation into CDKN2A expression and immune cell infiltration was conducted utilizing the TIMER database. Furthermore, Pearson correlation coefficients were computed to assess the relationship between CDKN2A expression and genes associated with immune checkpoint regulation.

### Correlation between expression of CDKN2A and sensitivity to immunotherapy

TIDE (Tumor Immune Dysfunction and Exclusion) was employed as a computational framework to assess the potential of tumor immune evasion in the gene expression profiles of the tumor samples. The TIDE score and the status of the tumor immune function for each tumor sample in the TCGA-COAD dataset were calculated in the TIDE database. The median value of the CDKN2A expression was used as the cutoff point to divide the CDKN2A gene into high and low expression groups, in order to predict the responsiveness of the CDKN2A gene expression level to immunotherapy.

## Gene correlation analysis of CDKN2A

To identify other genes associated with CDKN2A, we collected the top 10 genes directly related to CDKN2A expression from the STRING database. We used the GEPIA database to screen genes significantly coexpressed with CDKN2A. We conducted a Spearman correlation analysis, using the nonlog scale for calculation, and the log-scale axis for visualization. The HPA database (https://www.proteinatlas.org/) was also used to assess the expression of these genes in normal and tumor tissues.

## Results

### Identification of prognostic immune-related DEGs

**Fig. 1:**
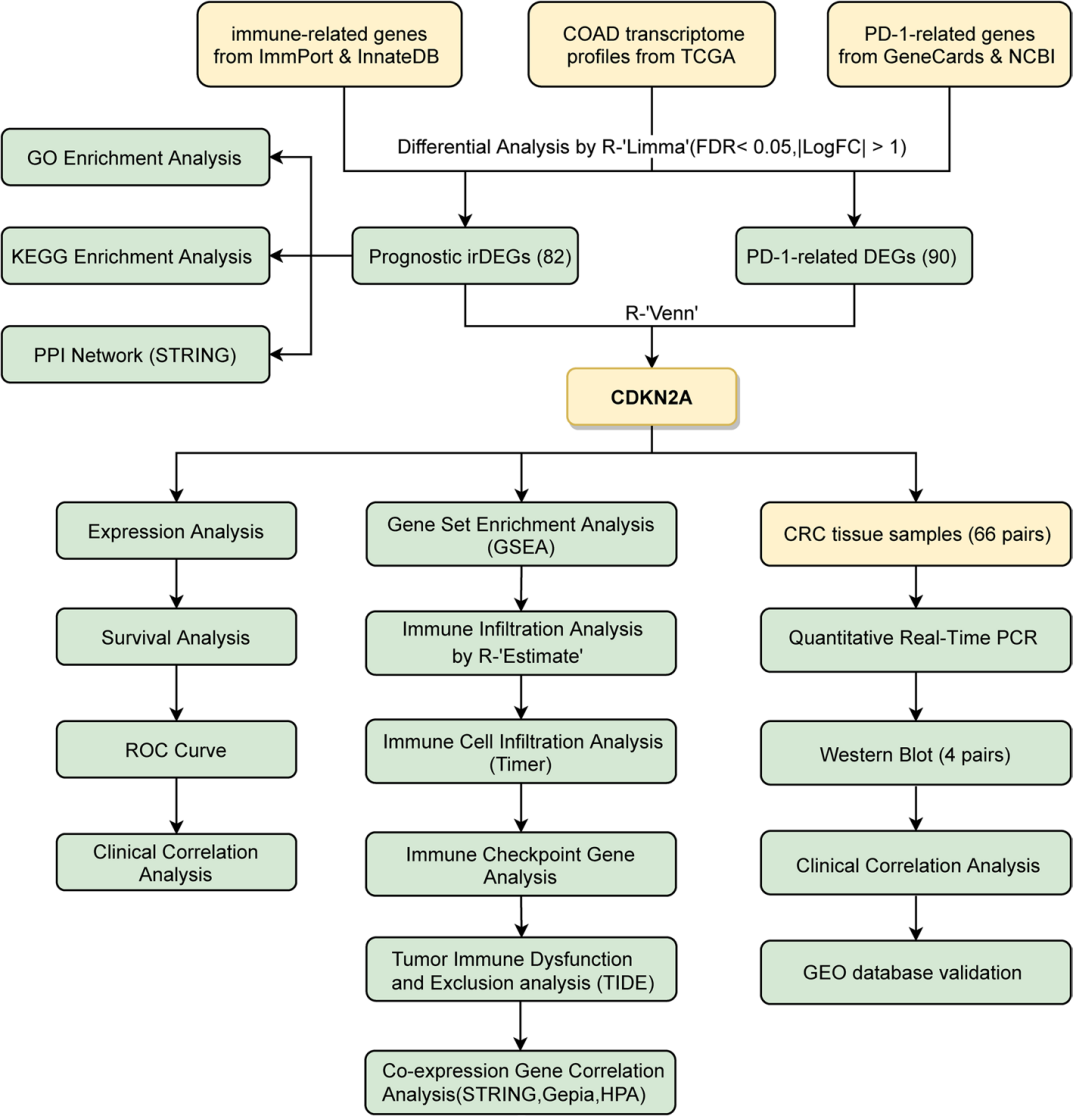
Study workflow. After obtaining data on colorectal cancer patients, immune-related genes, and PD-1-related genes from a database, differential analysis was conducted to identify prognosis-associated immune differential genes and PD-1-related genes, resulting in the discovery of the overlapping gene CDKN2A. Subsequently, gene expression analyses, survival and prognostic analyses, immune infiltration, enrichment analyses, and TIDE analysis were performed

**Fig. 2:**
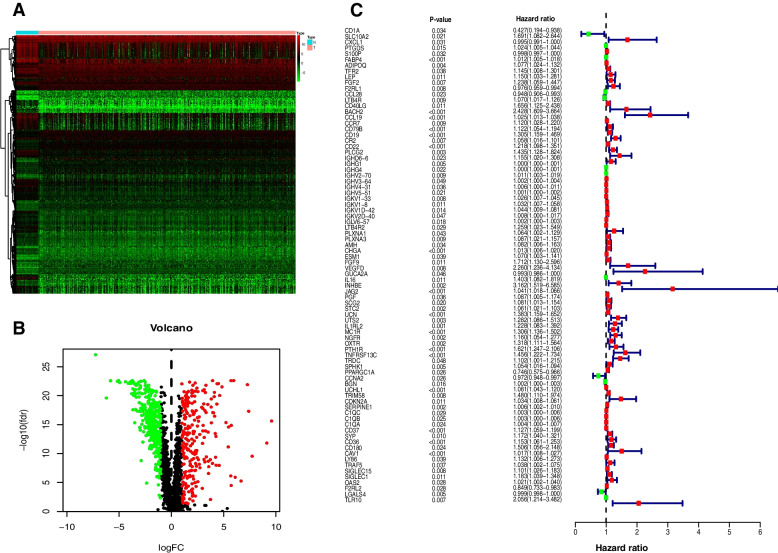
Identification of Prognostic irDEGs. **A** Heatmap of DEGs in COAD. Color scale green to red indicates low to high expression of DEGs. **B** The differential coexpression analysis identified 597 irDEGs. The red dots represent upregulated genes and the green dots represent downregulated genes. Black dots represent genes with no significant difference. **C** Forest plot showing the 82 prognostic irDEGs

Expression profiles were obtained from a total of 41 normal tissue samples and 473 colorectal cancer (CRC) samples sourced from the TCGA-COAD cohort (Fig. 1). We obtained 2,660 immune-related genes from the ImmPort and InnateDB datasets. The CRC tissues were found to be enriched for 597 genes (355 upregulated and 242 downregulated) as compared to adjacent normal tissues (Fig. 2A and B). Univariate Cox analysis was conducted to evaluate the irDEGs in COAD and determine the relevant impact of each gene individually on patient survival, identifying 82 irDEGs that were significantly associated with disease prognosis (Fig. 2C).

### GO/KEGG analysis and PPI network

**Fig. 3:**
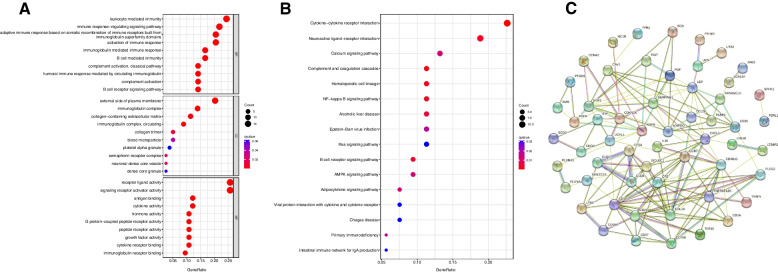
Functional enrichment analysis of prognostic irDEGs. **A** The top 10 most significantly enriched biological processes, cellular components, and molecular function terms from GO analysis. **B** The top 16 most significantly enriched pathways from KEGG analysis. **C **The PPI relationships determined among the prognostic indices

GO and KEGG analyses were performed on the 82 prognostic irDEGs. The most enriched biological processes (BP) were leukocyte-mediated immunity, immune response-regulating signaling pathway, adaptive immune response based on somatic recombination of immune receptors built from immunoglobulin superfamily domains, and immunoglobulin-mediated immune response. In terms of cellular components (CC), the prognostic irDEGs were mainly found in the external side of the plasma membrane, immunoglobulin complex, and collagen − containing extracellular matrix. The major functional categories related to molecular function (MF) were receptor ligand activity and signaling receptor activator activity (Fig. 3A).

The KEGG pathway enrichment analysis demonstrated that the prognostic irDEGs were significantly associated with cytokine-cytokine receptor interaction, neuroactive ligand − receptor interaction, and the calcium signaling pathway (Fig. 3B).

As shown in Fig. 3C, we constructed a protein–protein interaction (PPI) network and analyzed the internal relationships and connections among the prognostic irDEGs based on the STRING database.

### Differentially expressed PD-1-related genes in COAD and identification of overlapping genes

**Fig. 4:**
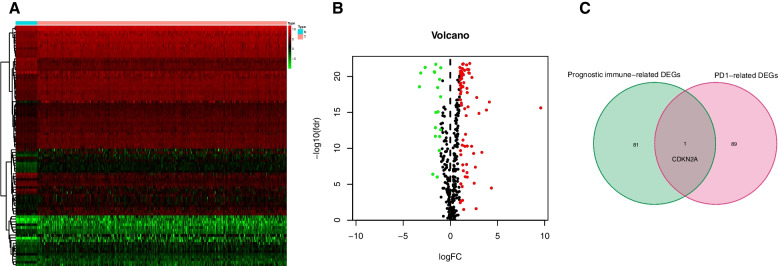
Differentially expressed PD-1-related genes in COAD. **A** Heatmap of the PD-1-related genes. **B** Volcano plot of the PD-1-related DEGs in COAD. **C** Screening the overlapping gene CDKN2A

A total of 9612 genes related to PD-1 were collected from GeneCards and NCBI databases, and analyzed in COAD. Among these PD-1-related genes, a comprehensive analysis revealed that a total of 90 genes exhibited differential expression patterns between COAD and normal tissue. Specifically, 71 genes were found to be upregulated, while 19 genes were downregulated in CRC tissue. Heatmap and volcano plot visualizations of the PD-1-related genes and DEGs were generated (Fig. 4A and B).

We conducted a comparative analysis of the PD-1-related genes and prognostic irDEGs, employing a threshold of |log2FC|> 1 and FDR < 0.05. Through this analysis, we successfully identified CDKN2A as a gene that is common to both sets (Fig. 4C).

### CDKN2A expression across cancers

**Fig. 5:**
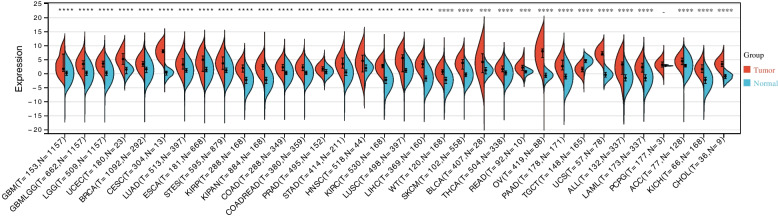
CDKN2A expression across cancers. *P < 0.05; **P < 0.01; ***P < 0.001

We analyzed CDKN2A expression in 34 human cancers using the UCSC database in order to explore possible effects of CDKN2A on cancer incidence and development. CDKN2A was significantly upregulated in 32 tumor types, while its expression was significantly decreased in TGCTs (Testicular Germ Cell Tumors) (tumor: 1.59 ± 1.22, normal: 4.30 ± 1.19, *p* = 3.7e-39; Fig. 5).

### Evaluation of CDKN2A expression and the prognostic significance of CDKN2A in COAD

**Fig. 6:**
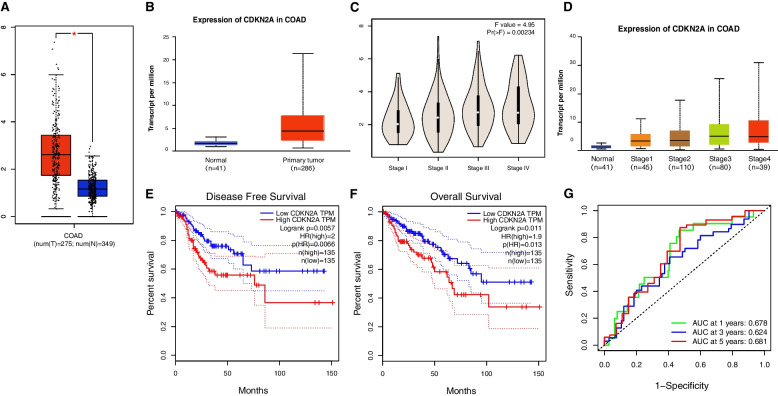
mRNA expression and clinical characteristics of CDKN2A in COAD. **A**, **B** CDKN2A mRNA expression compared between normal and tumor samples in the TCGA cohort by the GEPIA and UALCAN databases. **C**, **D** CDKN2A mRNA expression compared in different COAD stages. **E**, **F** Kaplan‒Meier curve of OS and DFS in COAD by GEPIA databases, based on CDKN2A mRNA expression. **G** The 1-, 3-, and 5-year AUC values

A comparative analysis of the CDKN2A gene expression was performed on cancer and normal samples using the GEPIA and UALCAN databases. In COAD, CDKN2A was highly expressed (Fig. 6A and B). We also found that CDKN2A expression is significantly different depending on the pathological stage of CRC (Fig. 6C and D). The prognostic value of CDKN2A for COAD was assessed and depicted using Kaplan–Meier curves. A high CDKN2A protein level was associated with shorter OS and DFS as compared to a low CDKN2A protein level (Fig. 6E and F). Moreover, survival status was predicted by ROC analysis, based on CDKN2A expression (Fig. 6G).

### The correlation between CDKN2A expression in CRC tissues and the clinicopathological features of CRC

**Fig. 7:**
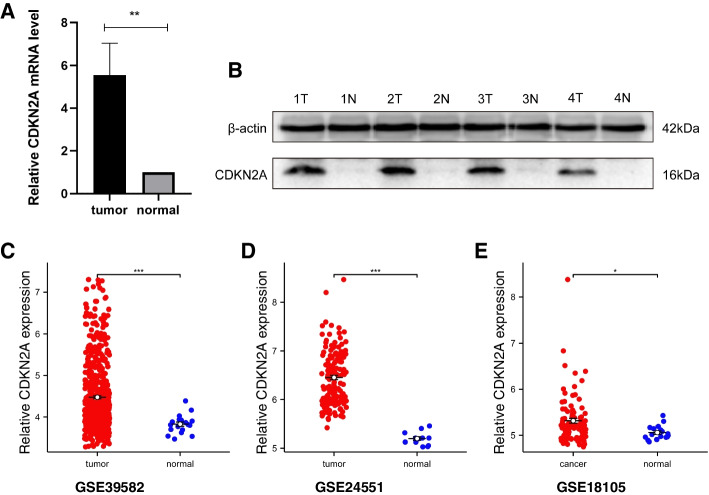
Expression of CDKN2A in CRC Tissues. **A** mRNA levels of CDKN2A in 66 pairs of colorectal cancer tissues and paired normal tissue. Data are shown as the mean ± SEM. **B** Western blot was used to measure the protein levels of CDKN2A in four pairs of colorectal cancer tumor tissues (T) and their paired normal tissues (N). Full-length blots/gels are presented in Supplementary Fig. 2. **C**-**E** In the GSE39582, GSE24551, and GSE18105 datasets, the levels of CDKN2A were upregulated in CRC tissues compared to normal tissues. *P < 0.05; **P < 0.01; ***P < 0.001

We conducted qRT-PCR analysis on 66 pairs of colorectal cancer and paired normal tissues, and randomly selected four pairs for Western Blot. To ensure the precision of the results, we utilized the GEO database to perform additional validation. Results indicated that, compared to normal tissues, the expression of CDKN2A mRNA and protein levels was higher in CRC tissues (Fig. 7A-E). In order to investigate the correlation between the expression levels of CDKN2A and the clinicopathological features of CRC patients, we divided the 66 CRC patients into two groups based on their relative expression of CDKN2A: a group with high expression (*n* = 34, > 1); and a group with low expression (*n* = 32, < 1). Our study revealed that the mRNA expression of CDKN2A did not exhibit any significant correlation with patient age, sex, body mass index (BMI), tumor location, tumor size, depth of infiltration, peri-intestinal lymph node metastasis, distant metastasis, P53 status, or Ki67 status. However, we observed a significant association between CDKN2A expression and tumor stage, as well as nerve invasion status (Table 1). By utilizing the GEO database, a Fisher test was conducted on the GSE40967 dataset, which consists of information from 585 colorectal cancer patients, to investigate the association between the high and low expression groups of CDKN2A with clinicopathological features. Our results demonstrated a significant association between CDKN2A expression and the status of TP53, KRAS, and BRAF.

**Table 1:** Correlation between CDKN2A expression levels and clinicopathological characteristics of colorectal cancer patients

Characteristics	Low(*N* = 34)	High(*N* = 32)	Total(*N* = 66)	*P* value
Gender				0.87
Female	10(15.15%)	11(16.67%)	21(31.82%)	
Male	24(36.36%)	21(31.82%)	45(68.18%)	
Age				0.66
≥ 65	23(34.85%)	19(28.79%)	42(63.64%)	
< 65	11(16.67%)	13(19.70%)	24(36.36%)	
BMI				0.2
≥ 25	6(9.09%)	11(16.67%)	17(25.76%)	
< 25	28(42.42%)	21(31.82%)	49(74.24%)	
Stage				0.03
I-II	12(18.18%)	21(31.82%)	33(50.00%)	
III-IV	22(33.33%)	11(16.67%)	33(50.00%)	
Tumor size(cm)				0.33
≥ 5	20(30.30%)	14(21.21%)	34(51.52%)	
< 5	14(21.21%)	18(27.27%)	32(48.48%)	
Tumor infiltration depth				0.61
T1-2	2(3.03%)	4(6.06%)	6(9.09%)	
T3-4	32(48.48%)	28(42.42%)	60(90.91%)	
Tumor location				0.32
Rectum	6(9.09%)	10(15.15%)	16(24.24%)	
Colon	28(42.42%)	22(33.33%)	50(75.76%)	
Distant metastasis				0.4
( +)	8(12.12%)	4(6.06%)	12(18.18%)	
(-)	26(39.39%)	28(42.42%)	54(81.82%)	
Peri-intestinal lymph node metastasis				0.2
( +)	18(27.27%)	11(16.67%)	29(43.94%)	
(-)	16(24.24%)	21(31.82%)	37(56.06%)	
deficient mismatch repair (dMMR)				0.73
( +)	4(6.06%)	2(3.03%)	6(9.09%)	
(-)	30(45.45%)	30(45.45%)	60(90.91%)	
P53				0.94
( +)	21(31.82%)	21(31.82%)	42(63.64%)	
(-)	13(19.70%)	11(16.67%)	24(36.36%)	
Vascular cancer embolism				0.47
( +)	10(15.15%)	6(9.09%)	16(24.24%)	
(-)	24(36.36%)	26(39.39%)	50(75.76%)	
Nerve Violation				0.03
( +)	21(31.82%)	10(15.15%)	31(46.97%)	
(-)	13(19.70%)	22(33.33%)	35(53.03%)	
Ki67%				
Mean ± SD	65.29 ± 11.61	61.25 ± 15.40	63.33 ± 13.63	
Median[min–max]	70.00[40.00,80.00]	65.00[20.00,90.00]	70.00[20.00,90.00]	

### Gene Set Enrichment Analysis (GSEA) of CDKN2A

**Fig. 8:**
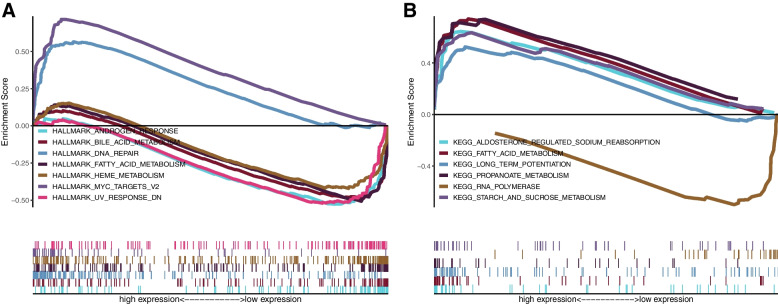
GSEA enrichment gene sets associated with CDKN2A expression in CRC: **A** Hallmark gene sets; **B** KEGG gene sets

The Gene Set Enrichment Analysis (GSEA) was conducted in order to explore the biological functions that are linked to the expression of CDKN2A. We found enrichment of several hallmark gene sets in the CDKN2A low-expression group, including the androgen response, heme metabolism, UV response downregulated, bile acid metabolism, and fatty acid metabolism (Fig. 8A). Conversely, the CDKN2A high-expression group showed a higher level of DNA repair and MYC target-v2 pathways (Fig. 8A). Furthermore, the results of KEGG gene set enrichment analysis revealed that the group with high expression of CKDN2A exhibited enrichment in various biological processes, including aldosterone-regulated sodium reabsorption, long-term potentiation, starch and sucrose metabolism, and fatty acid and propanoate metabolism (Fig. 8B). The CDKN2A low-expression group had significantly higher RNA polymerase pathway enrichment (Fig. 8B).

### Correlation between CDKN2A expression and characteristics of the tumor microenvironment in CRC

**Fig. 9:**
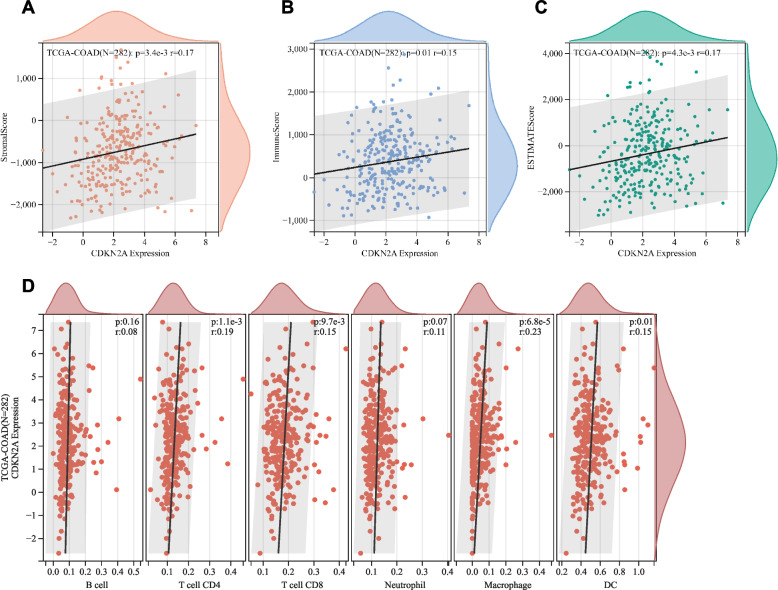
Associations between CDKN2A expression and tumor microenvironment characteristics in CRC. **A**-**C** Based on the ESTIMATE algorithm, there is a correlation between CDKN2A and stromal score, immune score, and ESTIMATE score in CRC samples. **D** Correlation of CDKN2A gene expression levels with the infiltration of six immune cell types by the TIMER database

ESTIMATE analysis was conducted to determine how CDKN2A affects the tumor microenvironment (TME) in colorectal cancer. ESTIMATE, immune score, and stromal score all showed positive correlations with CDKN2A expression (Fig. 9A-C). Moreover, we observed a significant association between CDKN2A expression and infiltration of macrophages, dendritic cells, CD4 T cells, and CD8 T cells (Fig. 9D). Furthermore, a positive correlation was observed between the expression of CDKN2A and various immune checkpoint genes in colorectal cancer, namely PDCD1, CTLA-4, and CD274 (Supplementary Fig. 1). Based on these results, we postulate that CDKN2A may be intrinsically linked to immune infiltration, immunological checkpoints, and the TME, thereby playing a crucial role in the regulation of T cells.

### Correlation between expression of CDKN2A and sensitivity to immunotherapy

**Fig. 10:**
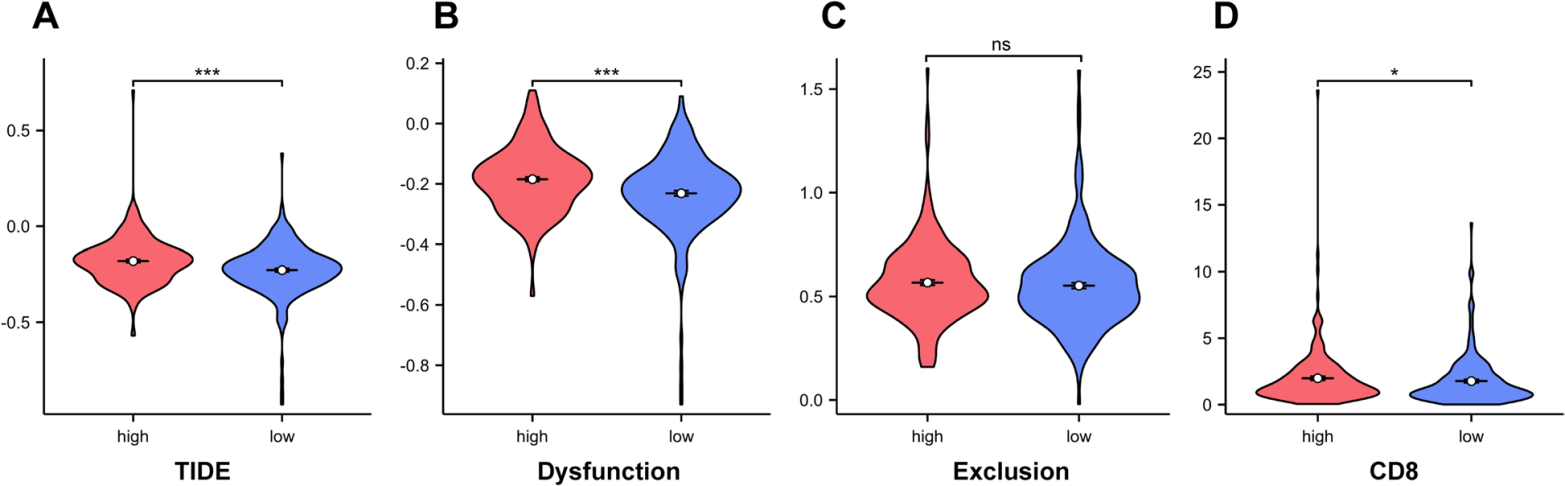
Correlation between expression of CDKN2A and sensitivity to immunotherapy through the TIDE database

To further explore the connection between the CDKN2A gene expression and tumor immunotherapy, we used the TIDE database to simulate the immune escape mechanism of tumor tissues and predict the response to immunotherapy. Our study revealed that the group with high CDKN2A expression had a much greater TIDE, Dysfunction, and CD8 scores than the group with low CDKN2A expression, implying that immunotherapy is more effective in treating colorectal cancers with high CDKN2A expression (Fig. 10).

### Gene correlation analysis of CDKN2A

**Fig. 11:**
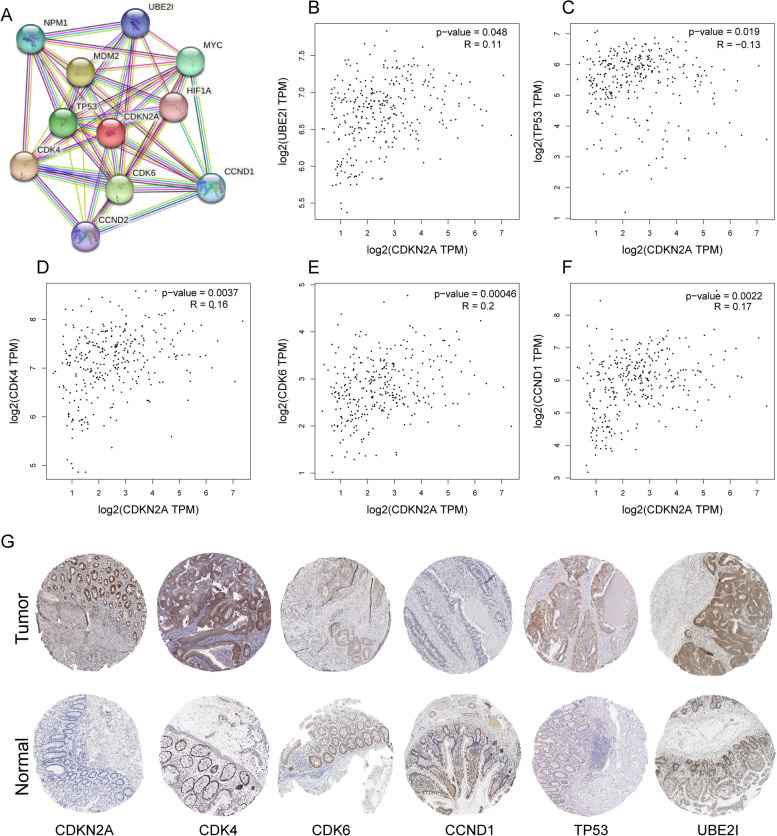
Identification of candidate proteins interacting with CDKN2A. **A** The top 10 candidate molecules that may interact with CDKN2A are shown. **B**-**F** The GEPIA database was used to screen for molecules that are significantly coexpressed with CDKN2A. **G** The HPA database was used to validate the differential expression of coexpressed molecules in CRC and normal colorectal tissue

For the purpose of exploring CDKN2A's involvement in immune-related pathways and CRC, we queried the STRING database and identified ten genes that interact directly with CDKN2A (Fig. 11A). We used the Gene Expression Profile Interaction Analysis (GEPIA) database to analyze the intensity of coexpression of these genes with CDKN2A and identified five genes that were significantly coexpressed with CDKN2A (Fig. 11B-F). Finally, we searched the HPA database for protein expression of CDKN2A and these coexpressed genes, and found that CDKN2A, UBE2I, CDK4, CDK6, TP53, and CCND1 were all highly expressed in CRC (Fig. 11G).

## Discussion

Immune checkpoint therapies, such as inhibitors targeting the PD-1/PD-L1 and CTLA-4 pathways, have ushered in a new phase in cancer treatment [8, 16]. However, the number of CRC patients who benefit from immunotherapy is limited [17, 18]. In this study, we screened for novel prognostic irDEGs and PD-1-related DEGs in CRC by differential analysis and univariate Cox analysis. We identified CDKN2A as a potential pivotal gene. Colorectal cancer tissues exhibited a significantly elevated level of CDKN2A mRNA expression compared to normal tissues, as confirmed through bioinformatic analysis and clinical observations. Mining the correlation between CDKN2A mRNA expression and clinicopathological features, including tumor status, distant metastasis, and pathological stage, as well as related molecular gene expression, is of great significance in diagnosing and treating CRC. Nevertheless, the present study did not yield any noteworthy disparities in the mRNA expression levels of CDKN2A and microsatellite instability among CRC patients with high and low expression, thereby indicating the necessity for further investigation within a more extensive CRC cohort to elucidate this association.

Immunotherapy aims to bolster natural anticancer immune defenses by mobilizing the host immune system antitumor immune responses against established tumor cells, thereby clearing tumor cells and inhibiting tumor cell migration and invasion [19, 20]. T cells and other molecules are recognized and interacted with by their surface molecules. Tumor immune response involves the action of CTLs (cytotoxic T lymphocytes), which are capable of killing tumor cells. The present process by which cytotoxic T lymphocytes (CTLs) eliminate tumor cells involves the secretion of various mediators and cytokines, including perforin, granzyme, and tumor necrosis factor (TNF) [21–23]. From tumor tissue, tumor-infiltrating lymphocytes (TILs) are isolated. The CD4 and CD8 molecules play a crucial role in the recognition of major histocompatibility complex (MHC) II and I molecules, respectively, and assist the T-cell receptor (TCR) in identifying bound antigens and participating in T-cell activation signaling [24–26]. Tumor-infiltrating immune cells are a significant predictor of survival in CRC patients [25, 27]. In the course of our investigation, it has been determined that CRC immune infiltration is potentially regulated by CDKN2A. Moreover, our research findings demonstrate a significant association between the expression of CDKN2A and the infiltration of CD4 T cells, CD8 T cells, and macrophages. These outcomes imply that CDKN2A might exert its influence on the immune microenvironment in colorectal cancer by means of immune cells or tumor-infiltrating lymphocytes (TILs), aligning with prior investigations [28, 29]. It is established that there is a strong correlation between the TME and tumorigenesis and progression [30, 31]. Consequently, the stromal score, immune score, and ESTIMATE score of colorectal cancer (CRC) tumors were computed, revealing a positive correlation between CDKN2A and the risk score, as well as the presence of stromal cells and immune cells. CDKN2A expression showed a strong correlation with the immune checkpoint molecules PDCD1, CD274, and CTLA4, so we hypothesized that ICIs might be effective in CRC patients with high CDKN2A expression. To validate our hypothesis, we conducted a TIDE analysis and found that those with a high CDKN2A expression had a greater response rate to immunotherapy and a higher chance of deriving benefit from it. Nevertheless, more experiments are needed to establish the role of CDKN2A in antitumor immunity.

The CDKN2A gene is a tumor suppressor located at the 9p21 locus, also called MTS1 (multiplex tumor suppressor 1), and encodes the proteins p16INK4a (p16) and p14ARF (p14). The phosphorylation of Rb proteins, leading to the inhibition of G1-phase cell proliferation, is a consequence of the interaction between p16INK4a and cell cycle protein-dependent kinase (CDK) 4/6 [32, 33]. Moreover, the protein p14ARF possesses the ability to interact with and degrade MDM2 (mouse double minute 2), consequently hindering the deactivation of p53 through ubiquitin-mediated proteolysis or transcriptional suppression [34]. The upregulation of the CDKN2A gene is notably observed in colorectal cancer, and this increased expression has been associated with unfavorable overall survival (OS) and disease-free survival (DFS) outcomes. The role CDKN2A plays as a tumor suppressor is contradicted by this finding. Possible explanations for this discrepancy may be attributed to changes in CDKN2A protein transcription and translation, as well as tumor heterogeneity. The features and functions of CDKN2A in CRC are still under debated [35]. CDKN2A is a highly prevalent gene mutation in human cancers [36, 37], with three distinct mechanisms identified as potential contributors to its inactivation: genomic deletions, point mutations, and hypermethylation of the CDKN2A promoter region CpG island [38–40]. Indeed, the hypermethylation of p16 and p14 has been found to be correlated with unfavorable prognosis, increased tumor aggressiveness, and the spread of metastases in individuals diagnosed with colorectal cancer [41–43].

Further investigation into the function of CDKN2A is necessary, so we conducted an analysis of a protein–protein interaction (PPI) network in the STRING database. From this analysis, we identified the top 10 potential interaction partners of CDKN2A, namely NPM1, UBE2I, CDK4, CDK6, TP53, MDM2, MYC, HIF1A, CCND1, and CCND2. These proteins play significant roles in cell cycle regulation and cancer pathways. Notably, UBE2I, CDK4, CDK6, TP53, and CCND1 exhibited the strongest coexpression with CDKN2A. Previous studies have highlighted the role of the p16INK4a-cyclin D1/Cdk4/CDK6-Rb axis and the P14ARF-MDM2-P53/TP53 axis in cancer [44–47]. However, the relationship between UBE2I (also called UBC9) and CDKN2A has been less explored. Several studies have focused on the interaction of ARF with the UBC9 SUMO-conjugating enzyme for stabilizing P53 [48, 49], this may provide guidance for our future research.

In conclusion, this study effectively discerned CDKN2A as a biomarker linked to immunity and PD-1 in colorectal cancer. The assessment of CDKN2A's expression and prognostic relevance in CRC was conducted comprehensively. Moreover, thorough examinations of immune infiltration and biological functionalities have demonstrated the potential of CDKN2A as a viable therapeutic target for colorectal cancer and may offer valuable insights for PD-1-mediated immunotherapy.

### Supplementary Information

**Additional file 1: Supplementary Figure 1. **Pearson correlation coefficients between CDKN2A expression and expression of immune checkpoint genes. **Supplementary Figure 2**. Corresponding full-length blot. **Supplementary Table 1**. Correlation between CDKN2A expression level and clinicopathologic characteristics of CRC patients in the GSE40967 dataset.

## Data Availability

The datasets analyzed during the current study are available in the TCGA database (https://portal.gdc.cancer.gov/), GEO database(https://www.ncbi.nlm.nih.gov/geo/, accession number: GSE39582, GSE24551, GSE18105, GSE40967), ImmPort database(https://www.immport.org/), InnateDB database (https://www.innatedb.ca/), GeneCards database (https://www.genecards.org/), NCBI database(https://www.ncbi.nlm.nih.gov/), UCSC database(https://xenabrowser.net/), STRING database(https://cn.string-db.org/), GEPIA database (http://gepia.cancer-pku.cn), UALCAN database (http://ualcan.path.uab.edu/analysis.html), GSEA (https://www.gsea-msigdb.org), Hallmark (h.all.v7.5.1.symbols.gmt), KEGG (c2.cp. Kegg.v7.5.1.symbols.gmt), TIMER database (http://timer.cistrome.org/),TIDE database (http://tide.dfci.harvard.edu/).

## Abbreviations

CRC: Colorectal cancer
PD-1: Programmed death protein 1
PD-L1: Programmed death protein ligand 1
COAD: Colon adenocarcinoma
GSEA: Gene Set Enrichment Analysis
CDKN2A: Cyclin-dependent kinase inhibitor 2A
FDA: U.S. Food and Drug Administration
TIL: Tumor-infiltrating lymphocyte
TME: Tumor microenvironment
TIDE: Tumor Immune Dysfunction and Exclusion
ICIs: Immune checkpoint inhibitors
irDEGs: Immune-related genes
FDR: False discovery rate
logFC: Log-fold change
GO: Gene Ontology
GEO: Gene Expression Omnibus
KEGG: Kyoto Encyclopedia of Genes and Genomes
PPI: Protein–protein interactions
TGCTs: Testicular Germ Cell Tumors
GEPIA: Gene Expression Profiling Interactive Analysis
OS: Overall survival
DFS: Disease-free survival
ROC: Receiver operating characteristic
AUC: Area under the curve
qRT‒PCR: Quantitative Real-Time PCR
NES: Normalized enrichment score
BP: Biological processes
CC: Cellular component
MF: Molecular function
BMI: Body mass index
UBE2I: UBC9, Ubiquitin Conjugating Enzyme E2 I
CDK4/6: Cell cycle protein-dependent kinase 4/6
TP53: Tumor protein 53
CCND1: Cyclin D1
CTLA-4: Cytotoxic T lymphocyte antigen 4
TNF: Tumor necrosis factor
MHC: Major Histocompatibility Complex
TCR: T-cell receptor
CTLs: Cytotoxic T lymphocytes
