# Supplementary material for: High expression of CDKN2A is associated with poor prognosis in colorectal cancer and may guide PD-1-mediated immunotherapy
Source: BMC Cancer. 2023 Nov 11;23:1097. doi: 10.1186/s12885-023-11603-w (PMC10638725; doi:10.1186/s12885-023-11603-w)
Supplement: Supplementary file 1 — Additional file 1: Supplementary Figure 1. Pearson correlation coefficients between CDKN2A expression and expression of immune checkpoint genes. Supplementary Figure 2. Corresponding full-length blot. Supplementary Table 1. Correlation between CDKN2A expression level and clinicopathologic characteristics of CRC patients in the GSE40967 dataset. [file 12885_2023_11603_MOESM1_ESM.docx]

**Supplementary Figure 1**. **Pearson correlation coefficients between CDKN2A expression and expression of immune checkpoint genes.**

**
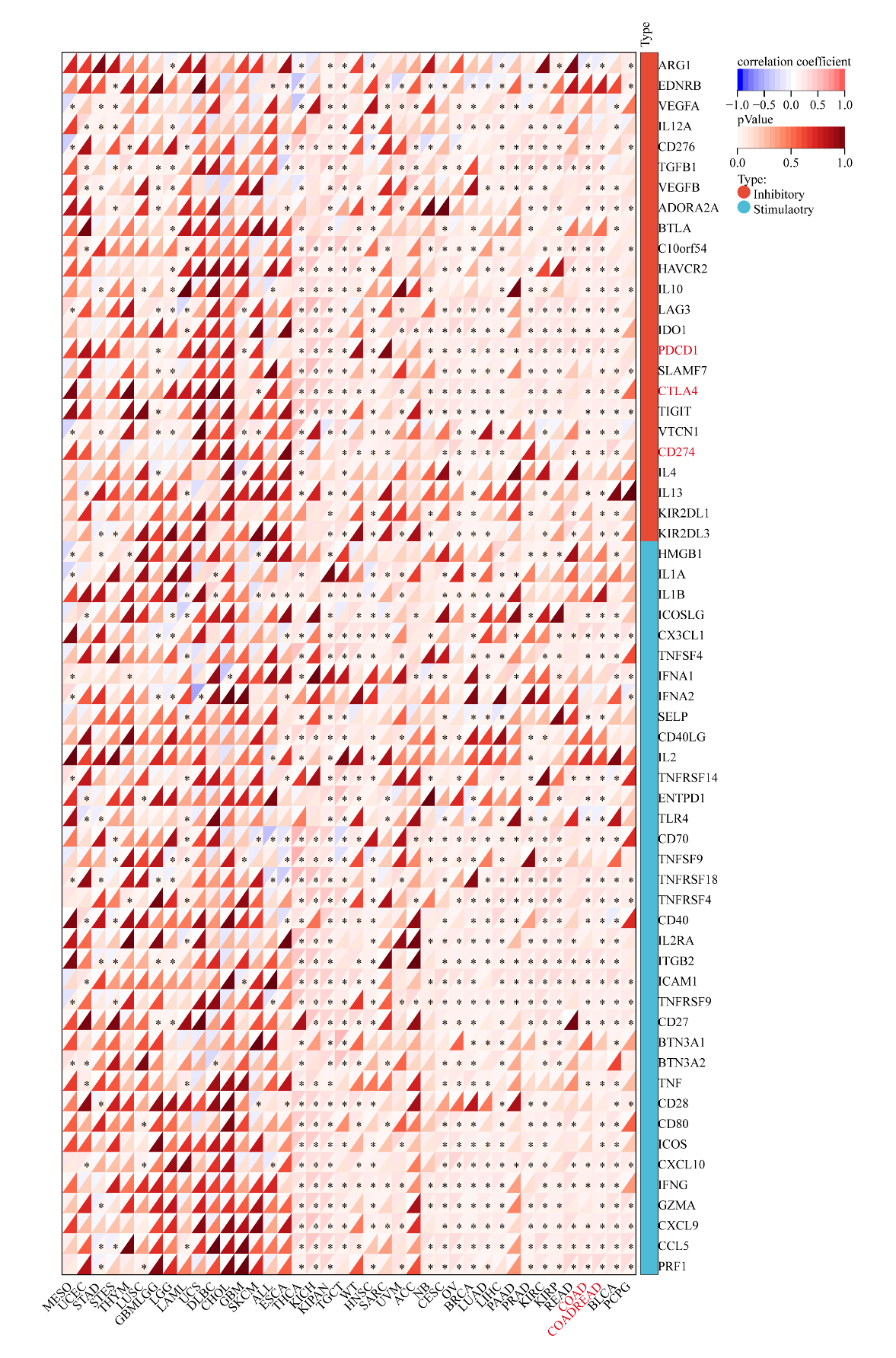
**

**Supplementary Figure 2.**
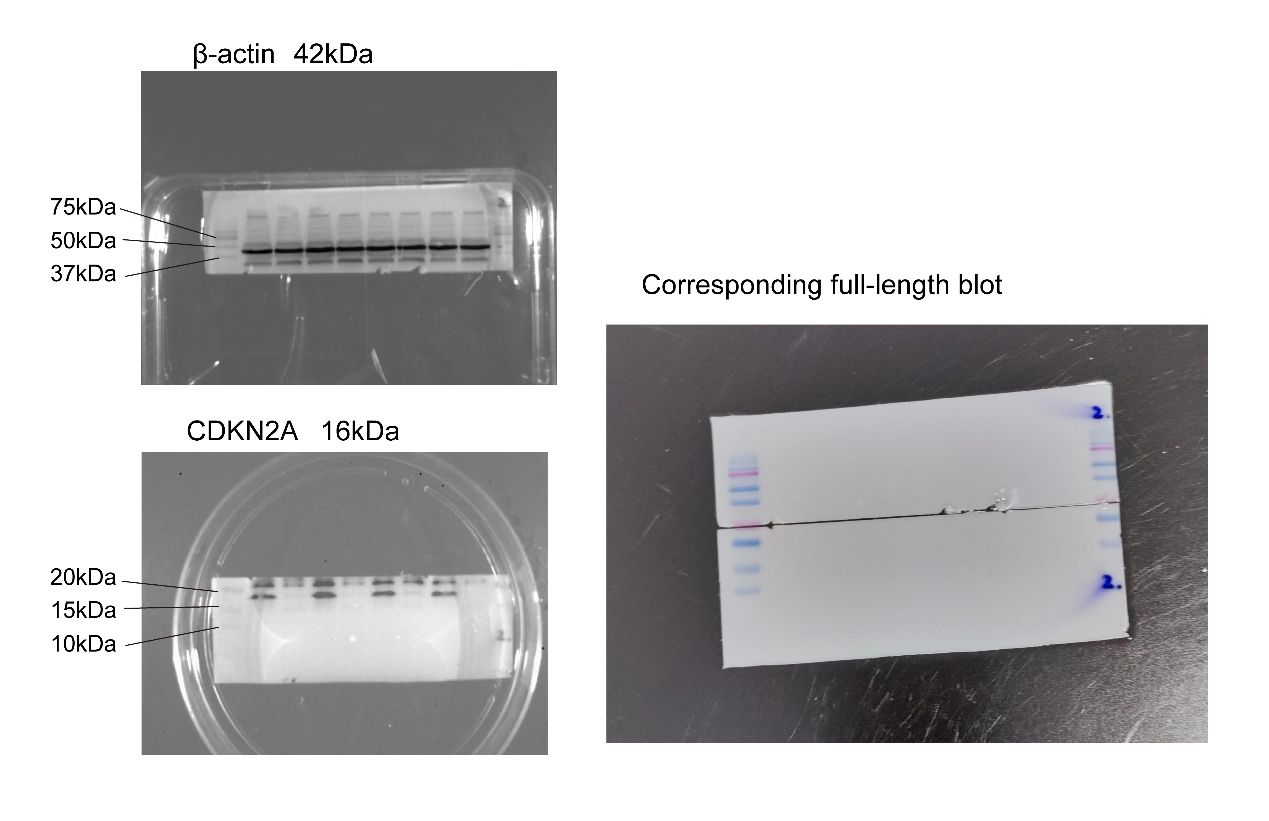


**Supplementary Table 1. Correlation between CDKN2A expression level and clinicopathologic characteristics of CRC patients in the GSE40967 dataset**.

| **Characteristics** | **Total (n = 585)** | **high (n = 293)** | **low (n = 292)** | **Statistic** | **p** |
| --- | --- | --- | --- | --- | --- |
| Gender, n (%) |  |  |  | χ²=2.377 | 0.123 |
| Female | 263 (44.96) | 141 (48.12) | 122 (41.78) |  |  |
| Male | 322 (55.04) | 152 (51.88) | 170 (58.22) |  |  |
| Age, n (%) |  |  |  | χ²=0.001 | 0.975 |
| ＜65 | 216 (36.92) | 108 (36.86) | 108 (36.99) |  |  |
| ≥65 | 369 (63.08) | 185 (63.14) | 184 (63.01) |  |  |
| Stage, n (%) |  |  |  | χ²=0.391 | 0.532 |
| S1-S2 | 315 (53.85) | 154 (52.56) | 161 (55.14) |  |  |
| S3-S4 | 270 (46.15) | 139 (47.44) | 131 (44.86) |  |  |
| T, n (%) |  |  |  | χ²=1.006 | 0.605 |
| T1-T2 | 65 (11.11) | 29 (9.90) | 36 (12.33) |  |  |
| T3-T4 | 498 (85.13) | 252 (86.01) | 246 (84.25) |  |  |
| N, n (%) |  |  |  | χ²=0.395 | 0.821 |
| N0 | 320 (54.7) | 157 (53.58) | 163 (55.82) |  |  |
| N1-N3 | 243 (41.54) | 124 (42.32) | 119 (40.75) |  |  |
| M, n (%) |  |  |  | χ²=0.508 | 0.776 |
| M0 | 499 (85.3) | 250 (85.32) | 249 (85.27) |  |  |
| M1 | 61 (10.43) | 29 (9.90) | 32 (10.96) |  |  |
| Tumor location, n (%) |  |  |  | χ²=0.003 | 0.959 |
| distal | 352 (60.17) | 176 (60.07) | 176 (60.27) |  |  |
| proximal | 233 (39.83) | 117 (39.93) | 116 (39.73) |  |  |
| MMR status, n (%) |  |  |  | χ²=1.854 | 0.396 |
| dMMR | 77 (13.16) | 33 (11.26) | 44 (15.07) |  |  |
| pMMR | 459 (78.46) | 235 (80.20) | 224 (76.71) |  |  |
| CIMP status, n (%) |  |  |  | χ²=0.777 | 0.678 |
| （-） | 420 (71.79) | 211 (72.01) | 209 (71.58) |  |  |
| （+） | 93 (15.9) | 49 (16.72) | 44 (15.07) |  |  |
| cin status, n (%) |  |  |  | χ²=3.301 | 0.192 |
| （-） | 112 (19.15) | 48 (16.38) | 64 (21.92) |  |  |
| （+） | 369 (63.08) | 194 (66.21) | 175 (59.93) |  |  |
| Tp53 status, n (%) |  |  |  | χ²=6.970 | 0.031 |
| M | 190 (32.48) | 110 (37.54) | 80 (27.40) |  |  |
| WT | 161 (27.52) | 73 (24.91) | 88 (30.14) |  |  |
| KRAS, n (%) |  |  |  | χ²=10.713 | 0.005 |
| M | 217 (37.09) | 123 (41.98) | 94 (32.19) |  |  |
| WT | 328 (56.07) | 158 (53.92) | 170 (58.22) |  |  |
| BRAF, n (%) |  |  |  | χ²=9.559 | 0.008 |
| M | 51 (8.72) | 23 (7.85) | 28 (9.59) |  |  |
| WT | 461 (78.8) | 245 (83.62) | 216 (73.97) |  |  |
